# A new algorithm for “the *LCS* problem” with application in compressing genome resequencing data

**DOI:** 10.1186/s12864-016-2793-0

**Published:** 2016-08-18

**Authors:** Richard Beal, Tazin Afrin, Aliya Farheen, Donald Adjeroh

**Affiliations:** Lane Department of Computer Science and Electrical Engineering, West Virginia University, Morgantown, WV USA

**Keywords:** Longest common subsequence, LCS, Longest previous factor, LPF, Compression, Biology, Genome resequencing

## Abstract

**Background:**

The longest common subsequence (LCS) problem is a classical problem in computer science, and forms the basis of the current best-performing reference-based compression schemes for genome resequencing data.

**Methods:**

First, we present a new algorithm for the LCS problem. Using the generalized suffix tree, we identify the common substrings shared between the two input sequences. Using the maximal common substrings, we construct a directed acyclic graph (DAG), based on which we determine the LCS as the longest path in the DAG. Then, we introduce an LCS-motivated reference-based compression scheme using the components of the LCS, rather than the LCS itself.

**Results:**

Our basic scheme compressed the *Homo sapiens* genome (with an original size of 3,080,436,051 bytes) to 15,460,478 bytes. An improvement on the basic method further reduced this to 8,556,708 bytes, or an overall compression ratio of 360. This can be compared to the previous state-of-the-art compression ratios of 157 (Wang and Zhang, 2011) and 171 (Pinho, Pratas, and Garcia, 2011).

**Conclusion:**

We propose a new algorithm to address the longest common subsequence problem. Motivated by our LCS algorithm, we introduce a new reference-based compression scheme for genome resequencing data. Comparative results against state-of-the-art reference-based compression algorithms demonstrate the performance of the proposed method.

## Background

Measuring similarity between sequences, be it DNA, RNA, or protein sequences, is at the core of various problems in molecular biology. An important approach to this problem is computing the longest common subsequence (*LCS*) between two strings *S*_1_ and *S*_2_, i.e. the longest ordered list of symbols common between *S*_1_ and *S*_2_. For example, when *S*_1_=abba and *S*_2_=abab, we have the following *LCS*s: abb and aba. The *LCS* has been used to study various areas (see [2, 3]), such as text analysis, pattern recognition, file comparison, efficient tree matching [4], etc. Biological applications of the *LCS* and similarity measurement are varied, from sequence alignment [5] in comparative genomics [6], to phylogenetic construction and analysis, to rapid search in huge biological sequences [7], to compression and efficient storage of the rapidly expanding genomic data sets [8, 9], to re-sequencing a set of strings given a target string [10], an important step in efficient genome assembly.

The basic approach to compute the *LCS*, between the *n*-length *S*_1_ and *m*-length *S*_2_, is via dynamic programming. Using *LCS* to denote the dynamic programming (DP) table, the basic formulation is as follows, given 0≤*i*≤*n* and 0≤*j*≤*m*:

$$LCS(i,j) = \left\{ \begin{array}{l} 0, \mathbf{if}\ \ i=0\ \vee\ j=0\\ 1 + LCS(i-1,j-1),\ \ \mathbf{if}\ \ S_{1}[\!i]=S_{2}[j]\\ \max \left\{LCS(i,j-1), LCS(i-1,j)\right\}, \\ \qquad \quad \mathbf{if}\ \ S_{1}[\!i] \neq S_{2}[j] \end{array}\right. $$

**Fig. 1:**
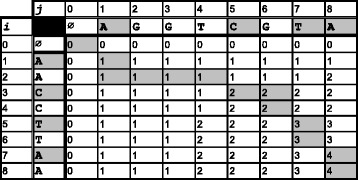
*LCS* dynamic programming table for *S*_1_=*A* *A* *C* *C* *T* *T* *A* *A* and *S*_2_=*A* *G* *G* *T* *C* *G* *T* *A*. A sample *LCS* trace (*ACTA*) is highlighted

The above computes the length of the *LCS* in the last position of the table (*LCS*(*n,m*)). As with the edit distance computation, the actual string forming the *LCS* can be obtained by using a trace back on the DP table. This requires *O*(*nm*) time and *O*(*nm*) space. The *LCS* matrix has some interesting properties: the entries in any row or in any column are monotonically increasing, and between any two consecutive entries in any row or column, the difference is either 0 or 1. An example *LCS* matrix and trace are shown in Fig. 1.

Alternatively, we can formulate the problem as a two-dimensional grid, where the goal is to find the minimal cost (or maximal cost, depending on the formulation) path, from the start position on the grid (typically, (0,0)), to the end position (*n,m*). Myers et al. [11] and Ukkonen [12] used this idea to propose a minimum cost path determination problem on the grid, where the path takes a diagonal line from (*i*−1,*j*−1) to (*i,j*) if *S*_1_[ *i*]=*S*_2_[ *j*] with cost 0, and takes a horizontal or vertical line with a cost of 1, corresponding respectively to insert or delete operations. Hunt and Szymanski [13] earlier used an essentially similar approach to solve the *LCS* problem in (*r*+*n*) log*n* time, with *n*≪*m*, where *r* is the number of pairwise symbol matches (*S*_1_[ *i*]=*S*_2_[ *j*]). When two non-similar files are compared, we will have *r*≪*nm*, or *r* in *O*(*n*), leading to a practical *O*(*n* log*n*) time algorithm. However, for very similar files, we have *r*≈*nm*, or an *O*(*nm* log*n*) algorithm. This worst-case occurs, for instance, when *S*_1_=*a*^*n*^ and *S*_2_=*a*^*m*^.

Hirschberg [14] proposed space-efficient approaches to compute the *LCS* using DP in *O*(*nm*) time and *O*(*n*+*m*) space, rather than *O*(*nm*). More recently, Yang et al. [15] used the observation on monotonically increasing values in the *LCS* table to identify the “corner points”, where the values on the diagonals change from one row to the next. The corners define a more sparse 2D grid, based on which they determine the *LCS*.

A generalization of the *LCS* problem is to find the *LCS* for a set of two or more sequences. This is the multiple longest common subsequence problem, which is known to be NP-hard for an arbitrary number of sequences [16]. Another interesting view of the *LCS* problem is in terms of the longest increasing subsequence (*LIS*) problem, suggested earlier in [17–19], and described in detail in [2]. The *LIS* approach also solves the *LCS* problem in *O*(*r* log*n*) time (where *m*≤*n*). In most practical scenarios, *r*<*nm*.

The *LCS* has been used in some recent algorithms to compress genome resequencing data [20, 21]. Compression of biological sequences is an important and difficult problem, which has been studied for decades by various authors [22–24]. See [9, 25, 26] for recent surveys. Most of the earlier studies focused on lossless compression because it was believed that biological sequences should not admit any data loss, since that would impact later use of the compressed data. The earlier methods also generally exploited self-contained redundancies, without using a reference sequence. The advent of high-throughput next generation sequencing, with massive datasets that are easily generated for one experiment, have challenged both compression paradigms.

Lossy compression of high-throughput sequences admitting limited errors have been proposed in [27, 28] for significant compression. With the compilation of several reference genomes for different species, more recent methods have considered lossless compression of re-sequencing data by exploiting the significant redundancies between the genomes of related species. This observation is the basis of various recently proposed methods for reference-based lossless compression [20, 21], whereby some available standard reference genome is used as the dictionary. Compression ratios in the order of 80 to 18,000 without loss have been reported [20, 21]. The *LCS* is the hallmark of these reference-based approaches. In this work, we first introduce a new algorithm for the *LCS* problem, using suffix trees and shortest-path graph algorithms. Motivated by our *LCS* algorithm, we introduce an improved reference-based compression scheme for resequencing data using the longest previous factor (*LPF*) data structure [29–31].

## Methods

### Preliminaries

A string *T* is a sequence of symbols from some alphabet *Σ*. We append a terminal symbol *$*∉*Σ* to strings for completeness. A string or data structure *D* has length- |*D*|, and its *i*th element is indexed by *D*[*i*], where 1≤*i*≤|*D*|. A prefix of a string *T* is *T*[1…*i*] and a suffix is *T*[*i*…|*T*|], where 1≤*i*≤|*T*|. The suffix tree (*ST*) on the *n*-length *T* is a compact trie (with *O*(*n*) nodes constructed in *O*(*n*) time [3]) that represents all of the suffixes of *T*. Suffixes with common prefixes share nodes in the tree until the suffixes differentiate and ultimately, each suffix *T*[ *i*…*n*] will have its own leaf node to denote *i*. A generalized suffix tree (*GST*) is an *ST* for a set of strings. A substring of *T* is *T*[ *i*…*j*], where 1≤*i*≤*j*≤*n*. The longest common subsequence is defined below in terms of length-1 common substrings.

#### *Definition 1.*

**Longest common subsequence (*LCS*)**: For the *n*-length *S*_1_ and *m*-length *S*_2_, the *LCS* between *S*_1_ and *S*_2_ is the length of the longest sequence of pairs $\mathcal {M}=\{m_{1},\ldots,m_{M}\}$, where *m*_*i*_=(*u,v*) such that *S*_1_[*m*_*h*_.*u*]=*S*_2_[ *m*_*h*_.*v*] for 1≤*h*≤*M* and *m*_*i*_.*u*<*m*_*i*+1_.*u* ∧ *m*_*i*_.*v*<*m*_*i*+1_.*v* for 1≤*i*<*M*.

### *LCS* algorithm

Below, we compute the *LCS* between *S*_1_ and *S*_2_ in the following way. (i) We use the *GST* to compute the common substrings (CSSs) shared between *S*_1_ and *S*_2_. (ii) We use the CSSs to construct a directed acyclic graph (DAG) of maximal CSSs. (iii) We compute *LCS* by finding the longest path in the DAG. Steps (i) and (iii) are standard tasks. For step (ii), we develop new algorithms and data structures.

### Computing the CSSs

We now briefly describe finding the common substrings (CSSs) between *S*_1_ and *S*_2_. In our *LCS* algorithm, for simplicity of discussion, we will only use CSSs of length-1.

Let $\mathcal {A}=\emptyset $. Compute the *GST* on *S*_1_*$*_1_∘*S*_2_*$*_2_, for terminals {*$*_1_,*$*_2_}. Consider a preorder traversal of the *GST*. When at depth-1 for a node *N*, let $\mathcal {S}=\emptyset $. During the preorder traversal from *N*, we collect in $\mathcal {S}$ all of the suffix index leaves descending from *N*, which represent the suffixes that share the same first symbol. Let $\mathcal {S}_{1}=\mathcal {S}_{2}=\emptyset $. For $s\in \mathcal {S}$, if *s*≤|*S*_1_|, then store *s* in $\mathcal {S}_{1}$. Otherwise, store *s* in $\mathcal {S}_{2}$. We represent all of our length-1 matches in the following structure: MATCH {*id, p1, p2*}. The *id* is a unique number for the MATCH, and *p*_1_ and *p*_2_ are respectively the positions in *S*_1_ and *S*_2_ where the CSS exists. Let *id*=2. Now, for each $s_{1}\in \mathcal {S}_{1}$, we create a new MATCH *m*=(*id*++,*s*_1_,*s*_2_) for each $s_{2}\in \mathcal {S}_{2}$. Store each *m* in $\mathcal {A}$.

The running time is clearly the maximum of the *GST* construction and the number of length-1 CSSs.

#### *Lemma 2.*

Say *n*= |*S*_1_| and *m*= |*S*_2_|, then computing the *η* CSSs of length-1 between *S*_1_ and *S*_2_ requires *O*(max{*n*+*m*,*η*}) time.

### DAG construction

Given all of the MATCHes found in $\mathcal {A}$, our task now is to construct the DAG for $\mathcal {A}$. For all paths of the DAG to start and end at a common node, we make MATCHes *S* and *E* to respectively precede and succeed the MATCHes in $\mathcal {A}$. (Let *S* have *id*=1 and *E* have $id=|\mathcal {A}+2|$ and then store *S* and *E* in $\mathcal {A}$.) The goal of the DAG is to represent all maximal CSSs between *S*_1_ and *S*_2_ as paths from *S* to *E*. We will later find the *LCS*, the longest such path.

In the DAG, the nodes will be the MATCH *id*s and the edges between MATCHes, say *m*_1_ and *m*_2_, represent that *S*_1_[ *m*_1_.*p*1]=*S*_2_[ *m*_1_.*p*2] is chosen in the maximal common subsequence followed by *S*_1_[ *m*_2_.*p*1]=*S*_2_[ *m*_2_.*p*2]. The DAG is acyclic because, by Definition 1, the *LCS* is a list of ordered MATCHes. Since we cannot choose $m_{i}\in \mathcal {M}$ and then $m_{h}\in \mathcal {M}$ with *h*<*i*, then no cycle can exist.

Our DAG construction, displayed in Algorithm 1, operates in the following way. We initialize the DAG *dag* by first declaring *dag*.*gr* of size $|\mathcal {A}|$, since *gr* will represent all of the nodes. All outgoing edges for say the node $N\in \mathcal {A}$ are represented by *dag*.*gr*[*N*.*id*][1…*dag*.*sz*[*N*.*id*]]. By setting *dag*.*sz*={0,…,0}, we clear the edges in our *dag*. Now, setting these edges is the main task of our algorithm.

We can easily construct the edges by assuming that there exists a data structure PREV *pv* that can tell us the set of parents for each node $a\in \mathcal {A}$. That is, we can call getPrnts(*pv,L*) to get the set of nodes *P* that *directly precede* MATCH $L\in \mathcal {A}$ in the final *dag*. By “directly precede”, we mean that in the final *dag*, there is connection from each *p*∈*P* to *a*, i.e. each *p* is in *series* with *a*, meaning that both *p* AND *a* are chosen in a maximal CSS. Further, no *p,p*2∈*P* can be in series with one another, and rather, they are in *parallel* with one another, meaning that either *p* OR *p*2 is chosen in a maximal common subsequence.

With *P*, we can build an edge from *a*2∈*P* to *a* by first allocating a new space in *dag*.*gr*[*a*2.*id*] by incrementing *dag*.*sz*[*a*2.*id*] and then making a directed edge from parent to child, i.e. *dag*.*gr*[*a*2.*id*][*dag*.*sz*[*a*2.*id*]]=*a*.*id*. After computing the incoming edges for each node $a\in \mathcal {A}$, the *dag* construction is complete.

#### *PREV* data structure

The simplicity of the DAG construction is due to the PREV *pv*, detailed here. The *pv* is composed of four attributes.

**HashMap** <**int,int** >*p*1. Suppose that all *a*.*p*1 values (for $a\in \mathcal {A}$) are placed on an integer number line. It is very unlikely that all *a*.*p*1 values will be consecutive and so, there will be unused numbers (gaps) between adjacent values. Since we later declare matrices on the MATCH *p*1 (and *p*2) values, these gaps will be wasteful. With a scan of the *a*.*p*1 values (say using a Set), we can rename them consecutively without gaps; these renamed values are found by accessing HashMap <int,int >*p*1 with the original *a*.*p*1 value.

**HashMap** <**int,int** >*p*2. This is the same as the aforementioned *p*1, but with respect to the *a*.*p*2 values.

**MATCH***tbl1*[][]. A fundamental data structure to support the getPrnts function is the *tbl*1, defined below.

##### *Definition 3.*

**Max Table w.r.t.***p*_1_(*tbl*1): Given the set of all MATCH values $\mathcal {A}$ and PREV *pv* on $\mathcal {A}$ (with *pv*.*p*1 and *pv*.*p*2), the *tbl*1[|*pv*.*p*1|][|*pv*.*p*2|] is defined such that each *tbl*1[*i*][*j*] is the $a\in \mathcal {A}$ with the **maximum***pv*.*p*1.*get*(*a*.*p*1)≤*i*, where *pv*.*p*2.*get*(*a*.*p*2)≤*j*. In the case that multiple such *a* exist, *tbl*1[ *i*][*j*] is the *a* with the **rightmost***pv*.*p*2.*get*(*a*.*p*2)≤*j*. If no such *a* exists, *tbl*1[ *i*][*j*]=*null*.

In other words, the *tbl*1[ *i*][*j*] stores the “closest” MATCH *a* with respect to the *p*_1_ values (i.e. we maximize *a*.*p*1 before *a*.*p*2). To construct *tbl*1, we first declare the table, *tbl*1[ |*pv*.*p*1|][ |*pv*.*p*2|] and initialize all elements *tbl*1[ *i*][*j*]=*null*, signifying that no MATCHes are found. Next, we insert each $a\in \mathcal {A}$ into the list by setting *tbl*1[ *pv*.*p*1.*get*(*a*.*p*1)][*pv*.*p*2.*get*(*a*.*p*2)]=*a*. Now, each *tbl*1[ *i*][*j*]=*null* needs to be set as the rightmost MATCH *m* with the maximum *m*.*p*1 in the subtable *tbl*1[ 1…*i*][ 1…*j*]. This is easily computed by first moving vertically in *tbl*1 and setting *tbl*1[ *i*][*j*]=*tbl*1[ *i*−1][*j*] if *tbl*1[ *i*][*j*]=*null* to propagate the maximum values vertically. Finally, we need to move horizontally in *tbl*1 and store in *tbl*1[ *i*][*j*] the rightmost *tbl*1[ *i*][ *v*](1≤*v*≤*j*) with the maximum *tbl*1[ *i*][ *v*].*p*1. This is done by a left-to-right scan of each row, comparing the adjacent elements, and setting *tbl*1[ *i*][ *v*]=*tbl*1[ *i*][ *v*−1] if *tbl*1[ *i*][ *v*−1].*p*1>*tbl*1[ *i*][ *v*].*p*1.

**MATCH***tbl2*[][]. The *tbl2* is the same as *tbl1* except that we define “closest” to mean that the *a.p2* value is maximized before the *a.p1*.

##### *Definition 4.*

**Max Table w.r.t.***p*_2_(*tbl*2): Given the set of all MATCH values $\mathcal {A}$ and PREV *pv* on $\mathcal {A}$ (with *pv*.*p*1 and *pv*.*p*2), the *tbl*2[ |*pv*.*p*1|][ |*pv*.*p*2|] is defined such that each *tbl*2[ *i*][*j*] is the $a\in \mathcal {A}$ with the **maximum***pv*.*p*2.*get*(*a*.*p*2)≤*j*, where *pv*.*p*1.*get*(*a*.*p*1)≤*i*. In the case that multiple such *a* exist, *tbl*2[ *i*][*j*] is the *a* with the **rightmost***pv*.*p*1.*get*(*a*.*p*1)≤*i*. If no such *a* exists, *tbl*2[ *i*][*j*]=*null*.

The construction of *tbl*2 is the same as *tbl*1, except that in the final horizontal scan, we compare *tbl*2[ *i*][*v*].*p*2 and *tbl*2[ *i*][ *v*−1].*p*2.







In terms of construction time, if we assume that adding and accessing HashMap entries are constant time operations, and the Set is implemented with a HashMap, then the PREV *pv* on $\mathcal {A}$ from the *n*-length *S*_1_ and *m*-length *S*_2_ is constructed in *O*(|*pv*.*p*1|×|*pv*.*p*2|) time. While *pv*.*p*1 and *pv*.*p*2 eliminate the gaps between the respective *p*1 and *p*2 values of $\mathcal {A}$, we have |*pv*.*p*1|∈*O*(*n*) and |*pv*.*p*2|∈*O*(*m*) in the very worst-case.

##### *Theorem 5.*

Given the *n*-length *S*_1_ and *m*-length *S*_2_, and the set of all MATCHes $\mathcal {A}$, PREV *pv* on $\mathcal {A}$ is constructed in *O*(*nm*) time.

#### getPrnts function

Given the PREV *pv* data structure on all MATCHes $\mathcal {A}$, we call getPrnts(*pv,L*) in line 11 of constructDAG to retrieve the set of parent MATCHes *P* of the MATCH $L\in \mathcal {A}$. Recall that these parents *P* of the MATCH *L* are all MATCHes that directly precede *L* in the DAG, i.e. each *p*∈*P* is in series with *L* and no *p,p*2∈*P* are in series with one another. Using *pv*, we can compute, for any MATCH $c\in \mathcal {A}$, two *direct parents* that are closest to *c* with respect to the *p*1 and *p*2 values.

##### *Definition 6.*

**Direct Parents**: Given the PREV *pv* on the MATCHes in $\mathcal {A}$ between the *n*-length *S*_1_ and the *m*-length *S*_2_, and a MATCH $c\in \mathcal {A}$, let *i*=*pv*.*p*1.*get*(*c*.*p*1) and *j*=*pv*.*p*2.*get*(*c*.*p*2). The *direct parent of c w.r.t. p1* is:

$${} d1 =\left\{ \begin{array}{l} \mathit{null},\ \text{if}\ i\leq 1\vee j\leq 1\vee i>|pv.p1|\vee j>|pv.p2| \\ pv.tbl1[\!i-1][j-1], \text{otherwise} \end{array}\right. $$

The direct parent of c w.r.t. p2 is:

$${} d2 =\left\{ \begin{array}{l} \mathit{null},\ \text{if}\ i\leq 1\vee j\leq 1\vee i>|pv.p1|\vee j>|pv.p2| \\ pv.tbl2[\!i-1][j-1], \text{otherwise} \end{array}\right. $$

The first getDPrnt in Algorithm 2 implements Definition 6 to return the direct parents for any MATCH say *L*∈*A*. In cases where we want to find the direct parent for a MATCH at a certain location in the *pv*.*tbl*1 or *pv*.*tbl*2, say *pv*.*tbl*1[ *i*][*j*] or *pv*.*tbl*2[ *i*][*j*], we overload getDPrnt.

The direct parents computation (getDPrnt) is the cornerstone of the getPrnts function. The following lemma, implemented in Algorithm 3, proves that the direct parents of *c* can be used to determine all parents of *c*.

##### *Lemma 7.*

Given $\mathcal {A}$, the MATCHes between *S*_1_ and *S*_2_, and a MATCH $c\in \mathcal {A}$, the two direct parents of *c* can be used to compute the set *P* with all parents of *c*.

##### *Proof.*

Let *d*1 and *d*2 be the direct parents of *c* (Definition 6). By Definition 3, *d*1 is a direct parent because it directly precedes *c* with the maximum *p*1 and the rightmost *p*2 value. Similarly by Definition 4, *d*2 is a direct parent of *c* because it directly precedes *c* with the maximum *p*2 and the rightmost *p*1 value. To find the remaining parents of *c*, we now find other MATCHes that precede *c*, which are also parallel with *d*1 and *d*2. There are three cases.

Case (a). When *d*1=*null*, then also *d*2=*null* since there cannot be another MATCH preceding *c*. Thus, *P*=*∅*.





Case (b). When *d*1=*d*2, the nearest parents to *c* are the same MATCH. There are only two types of MATCHes that are parallel with *d*1. First, we need to consider all MATCHes, say *m*1, with the same endpoint *m*1.*p*1=*d*1.*p*1 and *m*1.*p*2∈{1,2,…,*d*1.*p*2−1}. Second, we need to consider the MATCHes, say *m*2, with the same endpoint *m*2.*p*2=*d*1.*p*2 and *m*2.*p*2∈{1,2,…,*d*1.*p*1−1}. In the *LCS* computation, suppose that we chose, w.l.o.g., *m*1 (with *m*1.*p*2=*d*1.*p*2−2) instead of *d*1. Then, we cannot choose a MATCH *m*3 with *m*3.*p*1<*d*1.*p*1 and *m*3.*p*2=*d*1.*p*2−1. So, having any *m*1 or *m*2 parallel to *d*1 will only lead to suboptimal CSSs. Thus, only *P*={*d*1} is a parent of *c*.

Case (c). Otherwise, *d*1≠*d*2 and we have two different direct parents of *c*. Set *P*={*d*1,*d*2}. Let us collect the endpoints of *d*1 and *d*2: *i*1=*d*2.*p*1,*i*2=*d*1.*p*1,*j*1=*d*1.*p*2, and *j*2=*d*2.*p*2. What MATCH, say *m*3, is parallel to *d*1 and *d*2? By Definition 6, there cannot be any MATCH *m*3 directly preceding *c* with endpoints after *i*2 or *j*2. By (b), we do not need to consider other MATCHes with endpoints on either *d*1 or *d*2. So, all the *possible* MATCHes parallel to *d*1 and *d*2 are those with (*m*3.*p*1∈*w*∧*m*3.*p*2∈*x*), where *w*={*i*1+1,*i*1+2,…,*i*2−1} and *x*={*j*1+1,*j*1+2,…,*j*2−1}. To find such *m*3, we only need to find direct parents (by (b)), say *dd*1 and *dd*2, for a theoretical MATCH *m* with (*m*.*p*1∈*w*∧*m*.*p*2=*j*)∨(*m*.*p*1=*i*∧*m*.*p*2∈*x*). Then, when we have *i*1<*dd*1.*p*1<*i*2 and *j*1<*dd*1.*p*2<*j*2, this is a possible MATCH parallel with *d*1 and *d*2, which is also a possible parent of *c*, so we add *dd*1 to *P*. We do the same process for *dd*2.

Since we computed all the *possible* parents in *P*, additional processing on *P* is needed to ensure that no pair of MATCHes in *P* are in series; if any are in series, delete the MATCH furthest from *c*. With the *pv* and getDPrnt, this task is simple. We simply check the direct parents (say *dd*1 and *dd*2) for each *y*∈*P*, and remove *dd*1 if *dd*1∈*P* and remove *dd*2 if *dd*2∈*P*. □

### Computing the *LCS*

Since our *dag* has a single source *S* (and all paths end at *E*), the longest path between *S* and *E*, i.e. the *LCS*, is computed by giving all edges a weight of −1 and finding the shortest path from *S* to *E* via a topological sort [32].

### Complexity analysis

Our *LCS* algorithm: (i) finds the length-1 CSSs, (ii) computes the DAG on the CSSs, and (iii) reports the longest DAG path. Here, we analyze the overall time complexity.

#### Step (i)

First, we find (and store in $\mathcal {A}$) the *η* length-1 CSSs in *O*(max{*n*+*m*,*η*}) time by Lemma 2.

#### Step (ii)

We then construct the DAG *dag* on these $a\in \mathcal {A}$ with constructDAG. In constructDAG, we initially compute the newly proposed PREV *pv* data structure in *O*(*nm*) time by Theorem 5. After constructing *pv*, the computeDAG iterates through each $a\in \mathcal {A}$ and creates an incoming edge between the parents of *a* and *a*. So, computeDAG executes in time *O*(max{*nm*,*η*×*t*_getPrnts_}), where *t*_getPrnts_ is the time of getPrnts. The getPrnts running time is in *O*((*i*2−*i*1)+(*j*2−*j*1)), with respect to the local variables *i*1,*i*2,*j*1, and *j*2. However, it may be the case that *i*1=*j*1=1,*i*2=*n*, and *j*2=*m*, and so *O*(*n*+*m*) time is required by getPrnts. Below we formalize the worst-case result and the case for average strings from a uniform distribution.

##### *Lemma 8.*

For the *n*-length *S*_1_ and the *m*-length *S*_2_, the getPrnts function requires *O*(*n*+*m*) time.

##### *Lemma 9.*

For average case strings *S*_1_ and *S*_2_ with symbols uniformly drawn from alphabet *Σ*, the getPrnts function requires *O*(|*Σ*|) time.

##### *Proof.*

Since *d*1 and *d*2 are the direct parents of *c* (see Definitions 3, 4 and 6), and since the uniformness of *S*_1_ and *S*_2_ means that for any symbol say *S*_1_[*s*] we can find every *σ*∈*Σ* in *S*_2_[*s*−*Δ*…*s*+*Δ*] with *Δ*∈*O*(|*Σ*|), then (*i*2−*i*1)∈*O*(|*Σ*|) and (*j*2−*j*1)∈*O*(|*Σ*|). □

So, the overall constructDAG time follows.

##### *Theorem 10.*

Given $\mathcal {A}$, the length-1 MATCHes in the *n*-length *S*_1_ and the *m*-length *S*_2_, the constructDAG requires *O*(max{*nm*,*η*× max{*n,m*}}) time in the worst-case and *O*(max{*nm*,*η*×|*Σ*|}) on average.

#### Step (iii)

We find the *LCS* with a topological sort in time linear to the *dag* size [32], which cannot require more time than that needed to build the *dag* (see Theorem 10).

#### Summary

Overall, (i) and (iii) do not add to the complexity of (ii). Given the above, the overall running time is as follows.

##### *Theorem 11.*

The *LCS* between the *n*-length *S*_1_ and the *m*-length *S*_2_ can be computed in *O*(max{*nm*,*η*× max{*n,m*}}) time in the worst-case and *O*(max{*nm*,*η*×|*Σ*|}) on average.

### Compressing resequencing data

When data is released, modified, and re-released over a period of time, a large amount of commonality exists between these releases. Rather than maintaining all uncompressed versions of the data, it is possible to keep one uncompressed version, say *D*, and compress all future versions *D*_*i*_ with respect to *D*. We refer to *D*_*i*_ as the *target* and *D* as the *reference*. This idea is used to compress resequencing data in [20, 21], primarily using the *LCS*. The *LCS*, however, has two core problems with respect to compression. For very similar sequences, the *LCS* computation time is almost quadratic, or worse, potentially leading to long compression time. Secondly, the *LCS* may not always lead to the best compression, especially when some CSS components are very short.

Rather than focusing on the *LCS*, we consider the maximal CSSs that make up the common subsequences. To intelligently choose which of the CSSs are likely to lead to improved compression, we use the longest previous factor (*LPF*), an important data structure in text compression [33]. Consider compressing the target *T* with respect to the reference *R*; let *Z*=*R*∘*T*. Suppose we choose exactly |*T*| maximal-length CSSs, specifically, for *β*=*Z*[*i*…|*Z*|] we have *α*=*Z*[ *h*…|*Z*|] such that (1) CSSs *α*[1…*k*]=*β*[ 1…*k*] and (2) this is the maximal *k* for *h*<*i*, where |*R*|+1≤*i*≤|*Z*|. These *k*s are computed in the *LPF* data structure on *Z* at *LPF*[ *i*]=*k* and the position of this CSS is at *POS*[ *i*]=*h* [29]. (Note that *LPF* and *POS* are constructed in linear time [29–31].) The requirement that *h*<*i* suits dictionary compression and compressing resequencing data because the CSS beginning at *i* is compressed by referencing the same CSS at *h*, occurring earlier in target *T* or anywhere in the reference *R*. Our idea is to use the *LPF* and *POS* to *represent* or *encode* CSSs that make up the target *T* with tuples. We will then compress these tuples with standard compression schemes.

### Our compression scheme

We now propose a reference-based compression scheme which scans the *LPF* and *POS* on *Z* in a left-to-right fashion to compress *T* with respect to *R*. This scheme is similar to the LZ factorization [29], but differs in how we will encode the CSSs. Our contribution here is (1) using two files to compress *T*, (2) only encoding CSSs with length at least *k*, and (3) further compressing the resulting files with standard compression schemes.

Initially, the two output files, *triples* and *symbols*, are empty. Let *i*=|*R*|+1.

(!) If *LPF*[ *i*]<*k*, we simply encode the symbol; append the (say 1-byte) char *T*[ *i*−|*R*|] to *symbols* and increment *i*. Otherwise *LPF*[ *i*]≥*k*, so we will encode this CSS with the triple (*pT,pZ,l*), where *pT*=*i*−|*R*| is the starting position of the CSS in *T*, *pZ*=*POS*[*i*] is the starting position of the CSS in *Z*[ 1…*i*−1], and *l*=*LPF*[ *i*] is the length of the CSS. We write three long (say 4-byte integer) words *pT*, *pZ*, and *l* to *triples*. Since the triple encodes an *l*-length CSS, we set *i*=*i*+*l* to consider compressing the suffix following the currently encoded CSS. Lastly, if *i*≤|*Z*|, continue to (!).

The resulting files *triples* and *symbols* are binary sequences that can be further compressed with standard compression schemes (so, decompression will start by first reversing this process). The purpose of the *k* and the two files (one with byte symbols and one with long triples) is to introduce flexibility into the system and encode CSSs with triples (12 bytes) only when beneficial and otherwise, encode a symbol with a byte. For convenience, our implementation encodes each symbol with a byte, but we acknowledge that it is possible to work at the bit-level for small alphabets.

The decompression is also a left-to-right scan. Let *i*=1 and point to the beginning of *triples* and *symbols*.

(*†*) Consider the current long word *w*_1_ in *triples*. According to the triple encoding, this will be the position of the CSS in *T*. If *i*=*w*_1_, then we pick up the next two long words *w*_2_ and *w*_3_ in *triples*. We now know *T*[*i*…*i*+*w*_3_−1]=*Z*[*w*_2_…*w*_2_+*w*_3_−1]. Since we only have access to *R* and *T*[1…*i*−1], then we pick up each symbol of *Z*[*w*_2_…*w*_2_+*w*_3_−1] by picking up *R*[*j*] if *j*≤|*R*| and picking up *T*[*j*−|*R*|] otherwise, for *w*_2_≤*j*≤*w*_2_+*w*_3_−1. We next will consider *i*=*i*+*w*_3_. Else *i*≠*w*_1_, so we pick up the next char *c* in *symbols* since *T*[*i*]=*c*; we next consider *i*++. If *i*≤|*T*|, go to (*†*).

The compression and decompression algorithms are detailed in Algorithms 4 and 5, respectively.

## Results and discussion

We implemented the previously described compression scheme, selected and fixed parameter *k*, and ran our program to compress various DNA corpora. In this section, we describe the selection of *k* and our final results.

***Choosing parameter k***

Recall that the parameter *k* is a type of threshold used by our compression scheme to determine whether it is more beneficial to encode a symbol verbatim (that is, 1 byte) or encode a CSS as a triple (that is, 12 bytes). Specifically, our compression algorithm works on the *LPF* (which represents the CSSs of the *n*-length *T*) in a left-to-right fashion, selecting the leftmost CSS, say *T*[*i*…*i*+*l*−1] of length-(*LPF*[*i*]=*l*), and determining whether to encode that CSS as a triple [and then consider the next CSS (*T*[*i*+*l*…*i*+*l*+*LPF*[*i*+*l*]−1] of length- *LPF*[*i*+*l*])], or encode the first symbol (*T*[*i*]) [and then consider the next CSS (*T*[*i*+1…*i*+*LPF*[*i*+1]] of length- *LPF*[*i*+1])].

Obviously, it is better to encode a length-(*l*=1) CSS with a 1-byte symbol, rather than a 12-byte triple. It is clearly the case that for any CSS length 1≤*l*<12, it is better to encode the first symbol with 1-byte and take a *chance* that the next CSS to the right will be significantly larger. Why can we afford to take this *chance*? One *LPF* property, which also allows for an efficient construction of the data structure (see [29]), is that *LPF*[*i*+1]≥*LPF*[*i*]−1. That is, if we pass up on encoding the CSS at *i* of length-(*LPF*[ *i*]=*l*) as a triple, we can encode *T*[ *i*] as a symbol and (1) are guaranteed that there is at least a length-(*l*−1) CSS with a prefix of *T*[ *i*+1…*n*] and (2) the longest CSS common to a prefix of *T*[ *i*+1…*n*] is of length- *LPF*[ *i*+1], maybe even larger than *LPF*[ *i*]. Clearly, we want to encode most CSSs as triples to take advantage of the concise triple representation. Now, the question becomes: how large should we set *k*, such that we can afford to take a risk passing up length-(*l*<*k*) CSSs in hopes of finding even larger CSSs better suited as triples?

**Fig. 2:**
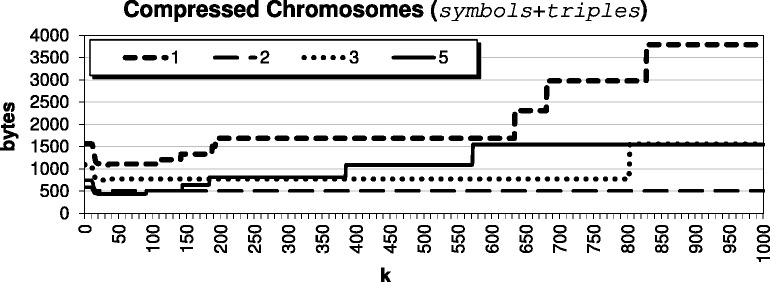
Total bytes needed by our algorithm to compress the *Arabidopsis thaliana* genome, i.e. file size sum of *symbols* and *triples*

**Fig. 3:**
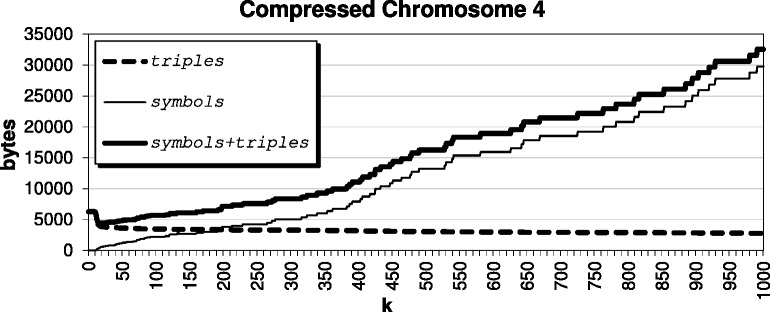
Compressing the *Arabidopsis thaliana* genome Chromosome 4

**Fig. 4:**
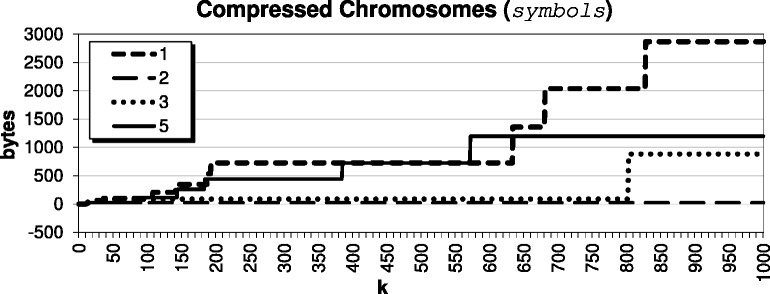
Size of the *symbols* file when compressing the *Arabidopsis thaliana* genome

**Fig. 5:**
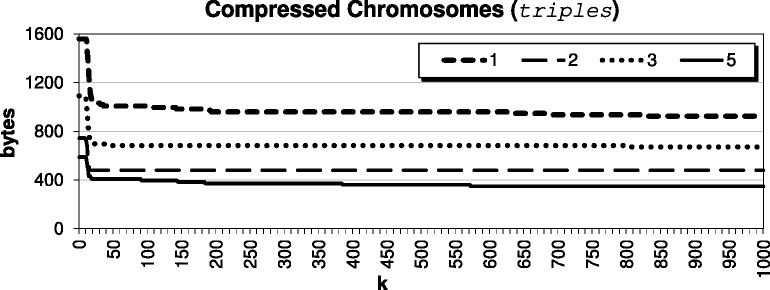
Size of the *triples* file when compressing the *Arabidopsis thaliana* genome

For this paper, we decided to select *k* by studying the impact of the parameter on our compressed results for the *Arabidopsis thaliana* genome, using target TAIR9 and reference TAIR8. The compression results for various *k* are shown in Fig. 2; since chromosome 4 does not compress as well as the others, we show it separately in Fig. 3 for improved visualization. For very small *k*<12, we have a result that basically encodes with triples only; when *k*=1, we are exclusively encoding CSSs as triples. We see that when *k* is roughly between 12 and 35, we are encouraging the algorithm to pass up encoding smaller CSSs as triples, which leads to the best compression result. The results stay competitive until say *k*≥100, where the algorithm becomes *too optimistic* and passes up the opportunity to encode smaller CSSs as triples in hopes that larger CSSs will exist. Further, we see from Fig. 4 that as *k* becomes large, it indeed becomes very expensive to pass up encoding these CSSs as triples. Also, we see from Fig. 5 that beyond say *k*=20, there is minimal compression savings. Thus, we want to balance the expensive *symbols* files with the space-savings from the *triples* files.

In Table 1, we show the best compression results and *k* for the *Arabidopsis thaliana* genome. Unless otherwise specified, our experiments below fix parameter *k* as 31, since it is the optimal *k* common to 4-of-5 of the *Arabidopsis thaliana* chromosomes and gives a competitive result for the remaining chromosome. This result follows intuition because *k* should be at least 11 and not too large, so that we can consider CSSs that are worthy of encoding.

**Table 1:** *Arabidopsis thaliana* genome: Optimal *k* for compressing chromosome *U* into the smallest *C* (in bytes)

*U*	*k*	|*C*|
1	31–35	1086
2	16–1578	504
3	24–39	746
4	18	4418
5	19–91	433

***Compression results***

Like [20, 21], we compress the *Arabidopsis thaliana* genome chromosomes in TAIR9 (target) with respect to TAIR8 (reference). In Table 2, we display the compression results. We see that all of our results are competitive with the GRS and GReEn systems, except for chromosome 4, which has the smallest average CSS length of about 326K. Nonetheless, we are able to further compress our results using compression schemes from 7-zip, with $\mathbb {L}$ and $\mathbb {P}$ respectively representing lzma2 and ppmd, to achieve even better compression.

**Table 2:** *Arabidopsis thaliana* genome: Results (in bytes) for compressing chromosome *U* into *C*

*U*	|*U*|	Our Scheme			GRS	GReEn
		|*C*|	$|\mathbb {L}(C)|$	$|\mathbb {P}(C)|$	[20]	[21]
1	30 427 671	1 086	963	1 037	**715**	1 551
2	19 698 289	504	584	605	**385**	937
3	23 459 830	**746**	759	803	2 989	1 097
4	18 585 056	4 555	2 507	3 156	**1 951**	2 356
5	26 975 502	**433**	502	520	604	618
Sum	119 146 348	7 324	**5 315**	6 121	6 644	6 559

Bold signifies the best result

In Table 3, we show results for compressing the genome *Oryza sativa* using the target TIGR6.0 and reference TIGR5.0. After compressing our algorithm’s output with lzma2 or ppmd, our results are better than both GRS [20] and GReEn [21]. Note that for each of the chromosomes 6, 9, and 12, our algorithm’s output is 12 bytes, better than that of GRS [20] (14 bytes) and GReEn [21] (482 bytes, 366 bytes, and 429 bytes respectively). When we compress our result with lzma2 or ppmd, the result is bloated since more bytes are needed. So, we can further improve the overall result by not compressing chromosomes 6, 9, and 12, and further, selecting the best such compression scheme for each individual chromosome. We acknowledge that additional bits would need to be encoded to determine which compression scheme was selected.

**Table 3:** *Oryza sativa* genome: Results (in bytes) for compressing chromosome *U* into *C*

*U*	|*U*|	Our Scheme			GRS	GReEn
		|*C*|	$|\mathbb {L}(C)|$	$|\mathbb {P}(C)|$	[20]	[21]
1	43 268 879	15 207	4 735	**4 551**	1 502 040	4 972
2	35 930 381	4 645	1 649	1 517	**1 409**	1 906
3	36 406 689	54 234	15 693	**15 556**	47 764	17 890
4	35 278 225	21 474	6 636	**6 432**	36 145	6 750
5	29 894 789	17 030	5 431	**5 359**	6 177	5 539
6	31 246 789	**12**	146	141	14	482
7	29 696 629	5 899	2 064	**1 972**	4 067	2 448
8	28 439 308	23 126	**8 794**	10 115	118 246	9 507
9	23 011 239	**12**	146	141	14	366
10	23 134 759	175 228	**49 713**	50 277	788 542	60 449
11	28 512 666	41 407	**13 006**	13 351	2 397 470	14 797
12	27 497 214	**12**	146	141	14	429
Sum	372 317 567	358 286	**108 159**	109 553	4 901 902	125 535

Bold signifies the best result

In Table 4, we show the compression results for the *Homo sapiens* genome, using KOREF_20090224 as the target and KOREF_20090131 as the reference. After compressing our computed *symbols* and *triples* files with lzma2, we see that most all of our results are better than GRS and GReEn. Recall in previous experiments that sometimes secondary compression with 7-zip does not improve the initial compression achieved by our proposed algorithm. For this genome, we exercise the flexibility of our compression framework. In Table 4, (*) indicates that the *M* chromosome was not further compressed with lzma2 due to the aforementioned reason. To indicate that *M* was not compressed, we will simply encode a length-25 bitstring (say 4-byte) header to specify whether or not the lzma2 was applied. There is no need to encode *k* in the header since it is a fixed value. Thus, the overall compressed files require 15,460,478 bytes, which is only slightly better than GRS and GReEn.

**Table 4:** *Homo sapiens* genome: Results (in bytes) for compressing chromosome *U* into *C*

*U*	|*U*|	Our Scheme		GRS	GReEn
		|*C*|	$|\mathbb {L}(C)|$	[20]	[21]
1	247 249 719	2 836 652	**1 082 859**	1 336 626	1 225 767
2	242 951 149	2 871 186	**1 050 170**	1 354 059	1 272 105
3	199 501 827	2 115 410	**790 444**	1 011 124	971 527
4	191 273 063	2 398 432	**910 898**	1 139 225	1 074 357
5	180 857 866	2 064 874	**764 458**	988 070	947 378
6	170 899 992	1 902 067	**710 355**	906 116	865 448
7	158 821 424	2 326 721	**844 194**	1 096 646	998 482
8	146 274 826	1 617 884	**617 996**	764 313	729 362
9	140 273 252	1 877 509	**704 205**	864 222	773 716
10	135 374 737	1 623 010	**617 633**	768 364	717 305
11	134 452 384	1 586 558	**604 901**	755 708	716 301
12	132 349 534	1 476 523	**566 997**	702 040	668 455
13	114 142 980	1 100 576	**399 527**	520 598	490 888
14	106 368 585	1 026 227	**377 695**	484 791	451 018
15	100 338 915	1 055 663	**398 720**	496 215	453 301
16	88 827 254	1 225 378	**443 009**	567 989	510 254
17	78 774 742	1 081 739	**396 371**	505 979	464 324
18	76 117 153	865 138	**320 361**	408 529	378 420
19	63 811 651	862 129	**320 789**	399 807	369 388
20	62 435 964	605 179	**229 418**	282 628	266 562
21	46 944 323	488 340	**180 096**	226 549	203 036
22	49 691 432	568 734	**205 244**	262 443	230 049
X	154 913 754	7 525 925	**2 494 884**	3 231 776	2 712 153
Y	57 772 954	1 343 260	**429 099**	592 791	481 307
M	16 571	151	151(*)	183	**127**
Sum	3 080 436 051	42 445 265	**15 460 474**	19 666 791	17 971 030

Bold signifies the best result

To improve this result, we exploit the difference between the *Homo sapiens* genome and those discussed earlier. That is, the *Homo sapiens* genome uses the extended alphabet {A, C, G, K, M, R, S, T, W, Y, a, c, g, k, m, n, r, s, t, w, y}. The observation is that, the alphabet size decreases roughly in half by converting to one character-case. Such a significant reduction in alphabet size will yield more significant redundancies identified by our compression algorithm. Our new *decomposition* method will *decompose* each chromosome into two parts: (1) the *payload* (*ρ*), representing the chromosome in one character-case, and (2) the *character-case bitstring* (*α*), in which each bit records whether the corresponding position in the target was an upper-case character. Next, we use our previously proposed algorithm to compress *ρ* into *C*^*ρ*^ and *α* into *C*^*α*^.

Table 5 shows compression via decomposition for the *Homo sapiens* genome. Note that the |*C*^*ρ*^|, i.e. compressed payload, column corresponds to the results reported in our initial work [1]. We observe that in various scenarios, the character-case of the alphabet symbol is not significant. For example, the IUB/IUPAC amino acid and nucleic acid codes use only upper-case letters (see http://www.bioinformatics.org/sms/iupac.html). Also, some environments and formats (such as FASTA) do not distinguish between lower-case and upper-case. According to the NCBI website for BLAST input formats (see http://blast.ncbi.nlm.nih.gov/blastcgihelp.shtml): “Sequences [in FASTA format] are expected to be represented in the standard IUB/IUPAC amino acid and nucleic acid codes, with these exceptions: lower-case letters are accepted and are mapped into upper-case; …” Further, some programs/environments use character cases for improved visualization, as is the case with the USC Genome Browser, which uses lower-case to show repeats from RepeatMasker and Tandem Repeats Finder (ftp://hgdownload.cse.ucsc.edu/goldenPath/hg38/chromosomes/README.txt).

**Table 5:** *Homo sapiens* genome: Results (in bytes) for compressing chromosome *U* via decomposition, i.e. compressing the payload (*ρ*) into *C* ^*ρ*^ and compressing the character-case bitstring *α* into *C* ^*α*^

*U*	|*U*|	Our Scheme					GRS	GReEn
		|*C* ^*ρ*^|	$|\mathbb {L}(C^{\rho })|$	|*C* ^*α*^|	$|\mathbb {L}(C^{\alpha })|$	$|\mathbb {L}(C^{\rho })|+|\mathbb {L}(C^{\alpha })|$	[20]	[21]
1	247 249 719	381 577	161 319	755 092	447 919	**609 238**	1 336 626	1 225 767
2	242 951 149	356 526	153 805	756 823	452 338	**606 143**	1 354 059	1 272 105
3	199 501 827	284 096	119 348	553 835	343 213	**462 561**	1 011 124	971 527
4	191 273 063	330 381	137 301	619 981	383 882	**521 183**	1 139 225	1 074 357
5	180 857 866	259 922	109 768	550 876	331 063	**440 831**	988 070	947 378
6	170 899 992	265 222	110 544	508 662	310 029	**420 573**	906 116	865 448
7	158 821 424	292 797	121 289	611 475	355 616	**476 905**	1 096 646	998 482
8	146 274 826	222 972	93 378	434 420	261 455	**354 833**	764 313	729 362
9	140 273 252	309 512	132 957	493 024	276 468	**409 425**	864 222	773 716
10	135 374 737	245 264	103 115	436 272	257 895	**361 010**	768 364	717 305
11	134 452 384	222 735	92 471	423 687	254 637	**347 108**	755 708	716 301
12	132 349 534	214 123	88 447	393 764	239 811	**328 258**	702 040	668 455
13	114 142 980	148 938	62 730	301 116	183 038	**245 768**	520 598	490 888
14	106 368 585	141 128	57 354	286 839	170 916	**228 270**	484 791	451 018
15	100 338 915	138 219	58 777	302 957	173 600	**232 377**	496 215	453 301
16	88 827 254	151 606	62 779	346 282	191 190	**253 969**	567 989	510 254
17	78 774 742	136 168	57 030	301 837	171 680	**228 710**	505 979	464 324
18	76 117 153	113 469	47 122	241 437	140 909	**188 031**	408 529	378 420
19	63 811 651	130 468	53 531	230 673	134 701	**188 232**	399 807	369 388
20	62 435 964	94 273	38 689	169 584	99 796	**138 485**	282 628	266 562
21	46 944 323	71 121	28 744	141 387	79 835	**108 579**	226 549	203 036
22	49 691 432	81 329	33 663	164 026	89 961	**123 624**	262 443	230 049
X	154 913 754	523 282	196 868	1 533 249	875 026	**1 071 894**	3 231 776	2 712 153
Y	57 772 954	152 464	57 002	300 287	153 582	**210 584**	592 791	481 307
M	16 571	64	64(*)	49	49(*)	**113**	183	127
Sum	3 080 436 051	5 267 656	2 178 095	10 857 634	6 378 609	**8 556 704**	19 666 791	17 971 030

Bold signifies the best result

Also, we see that further compressing the payload with lzma2 more than doubles the compression ratio. Interestingly, the payload (*ρ*) compresses much better than the character-case bitstring (*α*). Nonetheless, the compression via decomposition (in Table 5) yields a compression ratio of 360, a significant improvement over the 199 compression ratio when compressing the genome’s characters in their native character-case (in Table 4). As described earlier, we do not further compress chromosome *M* after initial coding for the symbols and triplets, and thus encode only a 4-byte header to remember this decision, given that the payload and character-case bitstring *k* values are fixed. Thus, 8,556,708 bytes are needed, which is an improvement over GRS and GReEn.

Theoretically, our compression scheme requires time linear in the length of the uncompressed text, since we perform one scan of the *LPF*, which is constructed in linear time via the suffix array *SA* [29]. For the *Arabidopsis thaliana* and *Oryza sativa* genomes, we ran our programs on a laptop; for the *Homo sapiens* genome, we ran our programs in an AWS EC2 m4.4xlarge environment. Consider, for example, the larger chromosomes of the *Homo sapiens* genome. For a payload (*ρ*), the *SA* construction required 2,376 sec and the *LPF* construction required 399 sec. Note that depending on the application, the *SA* and *LPF* may already be available. Given the *LPF*, our compression algorithm completed in less than one second. Decompression is also fast, and lightweight, since no data structures are required as parameters. Our future plan includes using more efficient *SA* and *LPF* constructions.

## Conclusions

We proposed a new algorithm to compute the *LCS*. Motivated by our algorithm, we introduced a new reference-based compression scheme for genome resequencing data using the *LPF*. For the *Arabidopsis thaliana* genome (originally 119,146,348 bytes), our scheme compressed the genome to 5315 bytes, an improvement over the best performing state-of-the-art methods (6644 bytes [20] and 6559 bytes [21]). For the *Oryza sativa* genome (originally 372,317,567 bytes), our scheme compressed the genome to 108,159 bytes, an improvement over the 4,901,902 bytes in [20] and the 125,535 bytes in [21]. We also experimented with the *Homo sapiens* genome (originally 3,080,436,051 bytes), which was compressed to 19,666,791 bytes and 17,971,030 bytes in [20] and [21], respectively. By applying our scheme via a decomposition approach, we compress the genome to 8,556,708 bytes, and if alphabet character-case is not significant, we compress the genome to 2,178,095 bytes. Further improvement can be obtained by choosing the *k* parameter for each specific chromosome, or each specific species.
